# Acoustoelasticity Analysis of Transient Waves for Non-Invasive *In Vivo* Assessment of Urinary Bladder

**DOI:** 10.1038/s41598-018-38445-y

**Published:** 2019-02-21

**Authors:** Mahdi Bayat, Saba Adabi, Viksit Kumar, Adriana Gregory, Jeremy Webb, Max Denis, Baehyung Kim, Aparna Singh, Lance Mynderse, Douglas Husmann, Azra Alizad, Mostafa Fatemi

**Affiliations:** 1 Department of Physiology and Biomedical Engineering, Mayo Clinic College of Medicine & Science, Rochester, MN USA; 2 Depratment of Radiology, Mayo Clinic College of Medicine & Science, Rochester, MN USA; 3 School of Engineering and Applied Science, University of District of Columbia, Washington DC, USA; 4 Depratment of Urology, Mayo Clinic College of Medicine & Science, Rochester, MN USA

## Abstract

A non-invasive method for measurement of the bladder wall nonlinear elastic behavior is presented. The method is based on acoustoelasticity modeling of the elasticity changes in bladder tissue modulus at different volumetric strain levels. At each volume, tissue strain is obtained from the real-time ultrasound images. Using acoustic radiation force, a transient Lamb wave is excited on the bladder wall and instantaneous modulus of shear elasticity is obtained from the 2-D Fourier analysis of the spatial-temporal dispersion maps. Measured elasticity and strain data are then used in an acoustoelasticity formulation to obtain the third order elastic coefficient, referred to as *nonlinearity parameter A*, and initial resting elasticity *μ*_0_. The method was tested in *ex vivo* porcine bladder samples (N = 9) before and after treatment with formalin. The estimated nonlinearity parameter, *A*, was significantly higher in the treated samples compared to intact (*p* < 0.00062). The proposed method was also applied on 16 patients with neurogenic bladders (10 compliant and 6 non-compliant subjects). The estimated nonlinearity parameter *A* was significantly higher in the non-compliant cases compared to the compliant (*p* < 0.0293). These preliminary results promise a new method for non-invasive evaluation of the bladder tissue nonlinearity which may serve as a new diagnostic and prognostic biomarker for management of the patients with neurogenic bladders.

## Introduction

Bladder is a complex organ and its bidirectional storing-voiding functionality is closely tied to the mechanobiology of its multi-layer wall. The main function of bladder is to control micturition and to store urine at low pressure^1^. During the filling–voiding procedure, bladder undergoes massive deformations which requires a unique stretching and recoiling capability^2^. Hence, characterization of the bladder wall mechanical properties is crucial in diagnosis of poor compliance that can lead to lower tract infection and incontinence. Urodynamic studies (UDS) have been conventionally used to indirectly assess bladder mechanical properties via artificial filling and observing changes in pressure^3^. UDS consists of gradually filling the bladder via a catheter at a predetermined rate and measuring detrusor pressure (pressure across the bladder wall). The UDS results are reported in terms of pressure-volume charts from which physicians are able to assess bladder compliance defined as maximum volume changes over maximum pressure changes^3^. Additionally, pressure-volume curves are closely observed as unexpected changes in the pressure rate at different volumes can be indicative of abnormalities such as bladder overactivity. While UDS is a well-accepted procedure it has some limitations like its invasive nature, fear of infection and some degree of subjectivity in the interpretation of the results^3^. Additionally, methods that can directly estimate bladder wall mechanical properties (instead of changes in the pressure) can provide additional information which may be used as biomarkers associated with the abnormal bladder.

Ultrasound bladder vibrometry (UBV) has emerged as a noninvasive method in characterization of the bladder tissue elasticity using acoustic radiation force and Lamb wave for modeling the transient waves^4,5^. When tested in a group of patients, UBV findings were shown to strongly correlate with the UDS results which is considered as the gold standard for assessment of the bladder compliance^6,7^. These studies have shown an increasing trend in the bladder elasticity with increasing fluid volume, which is an indication of a nonlinear behavior. Hence, in addition to the linear modulus of elasticity, nonlinearity can be investigated as a potential biomarker of the bladder wall mechanical properties.

Transient waves in pre-stressed soft tissue have been analyzed to determine the tissue hyperelastic parameters. By observing strain-stress behavior, previous studies on breast tissues showed that nonlinear parameter can be an appropriate supplement to improve diagnosis^8–11^. Primarily, Gennisson *et al*. presented a theoretical approach to investigate the nonlinear behavior of quasi-incompressible soft solids^12^. In other study, Oberai *et al*. studied the nonlinear variation of strain distribution with the overall strain, the variation of the secant modulus with overall applied strain and the distribution of the nonlinear parameter in a nonlinear hyperelastic model of the breast tissue^10^. Krouskop *et al*.^8^ investigated the breast tissue nonlinear response using a mechanical compression loading and concluded that the elastic modulus of different tissues could be better distinguished utilizing higher levels of strain. The shear wave velocity measurement and determination of the acoustoelasticity (AE) phenomenon was reported previously^12–16^ and was applied to transplanted kidneys (for a pool of patients) by Syversveen *et al*.^17^. Recently, Aristizabal *et al*.^18^ assessed the feasibility of estimating nonlinear shear modulus in the kidney using shear wave elastography combined with an external compression in an *ex vivo* study. In addition, Latorre *et al*. proposed a method to combine static elastography and shear wave elastography to derive the nonlinear shear modulus by applying the AE theory in soft solids^14^. In line with Ossa’s work, Bernal *et al*.^13^ studied the nonlinear characteristics of breast tissues and demonstrated nonlinear modulus as a promising differentiator between malignant lesions and the healthy tissue. An inverse approach to measure A was adopted based on measurement of strain field using static elastography. By gradually increasing the compression and measuring the incremental strain and shear wave velocities, the relationship between the stress and the shear wave velocity can be utilized to determine the nonlinear parameter.

In this study, we use the AE theory to quantify the nonlinearity parameter, A, for bladder tissue modelled as a thin walled soft tissue. To measure mechanical properties of such a medium, the variation of the phase velocity of guided waves in the structure is a key factor to be addressed. In recent studies, the guided wave formulation is adopted in ultrasound elastography of thin-walled soft tissues^4,19,20^. The main hypothesis of this study is the bladder tissue can be considered as a pre-stressed thin shell with nonlinear elasticity behavior. Li *et al*.^21^ have recently shown that changes in nonlinearity parameter *A* can be detected via analysis of transient guided waves in pre-stressed medium. Using a simplistic cumulative stress-strain model, an earlier *ex vivo* study showed that nonlinear parameter changes drastically in aberrant tissue compared to the intact ones^22^.

In this study, we use the AE theory to measure *A* for bladder tissue using instantaneous measurement of the shear elasticity using UBV method. These modulus readings along with measured uniaxial strain values are used in an AE constitutive equation to find the nonlinearity parameter *A* and resting shear modulus *μ*_0_. We show that the nonlinearity parameter alone can precisely differentiate between the intact and aberrant *ex vivo* bladder samples treated with formalin. Additionally, we apply this method in a group of patients with diagnosed bladder condition based on UDS to assess the utility of nonlinearity parameters *A* in differentiation of compliant and noncompliant bladders. The outcomes of this study promise a new parameter for assessment of the bladder mechanical state which might be relevant in noninvasive diagnosis of different diseases and conditions affecting mechanical properties of the bladder wall.

## Results

### *Ex Vivo* Study

**Figure 1:**
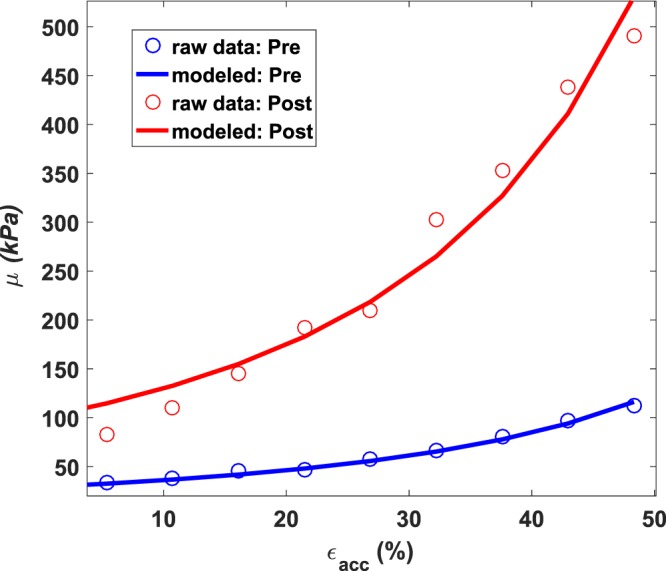
Elasticity measured by UBV at each strain (in kPa versus % strain) level for pre (blue) and post (red) formalin treated bladder and their resulting nonlinear fits used to estimate the nonlinear shear modulus A.

Nine freshly excised *ex vivo* porcine whole bladder samples were acquired for this study. The spatio-temporal map of the tissue motion in response to acoustic radiation force along the bladder wall (one direction) obtained from *ex vivo* experiment on porcine bladder sample. Using two-dimensional Fourier transformation, the Lamb wave dispersion data based on k space calculations is obtained and at each point along the ridge the best Lamb wave fit has been found. It was assured the analytic fit using Lamb model is in good agreement with the experimental data for a large range of frequencies. Figure 1 shows the shear elasticity variations as a function of accumulated strain, ${{\epsilon }}_{acc}$, measured by UBV before and after formalin treatment for one sample. As it can be seen, in the intact bladder, the shear elasticity gradually increases with strain. These changes, however, present much higher rates after formalin treatment which is indicative of significant changes in the nonlinear elasticity parameter *A*. The AE model provided a close fit for experimental data with a mean fitting *r*^2^ value of 0.96 ± 0.096 and 0.93 ± 0.096 for pre and post samples respectively.

**Figure 2:**
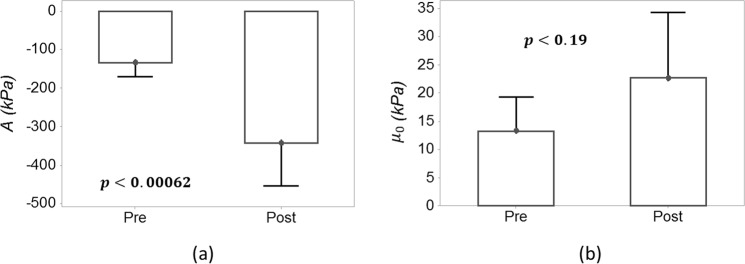
Box plot of nonlinearity parameter $A$ and ${\hat{\mu }}_{0}\,\,$ in intact (pre) and formalin treated (post) from 9 *ex vivo* bladder samples.

Figure 2(a,b) show a box-plot of the estimated *A* and *μ*_0_ in intact and aberrant bladders in 9 samples respectively. The absolute median nonlinearity parameter in the aberrant bladders was significantly higher than the absolute mean nonlinearity parameter in the intact bladders (*p* < 0.00062). The median estimated resting shear elasticity, *μ*_0_, was higher in the formalin-treated samples but this difference was not significant between the two groups (*p* < 0.19).

### *In Vivo* Study

**Figure 3:**
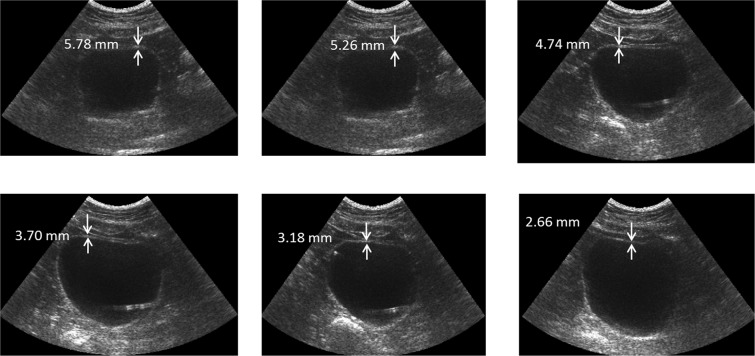
Changes in bladder thickness during UDS filling in a patient.

The clinical UDS study on 16 patients revealed 10 subjects as compliant and 6 subjects as non-compliant. Figure 3 depicts the volumetric changes in the bladder for a patient undergoing concurrent UDS and UBV. It can be observed that as the bladder volume increases, the wall becomes thinner which translates into higher levels of strain.

**Figure 4:**
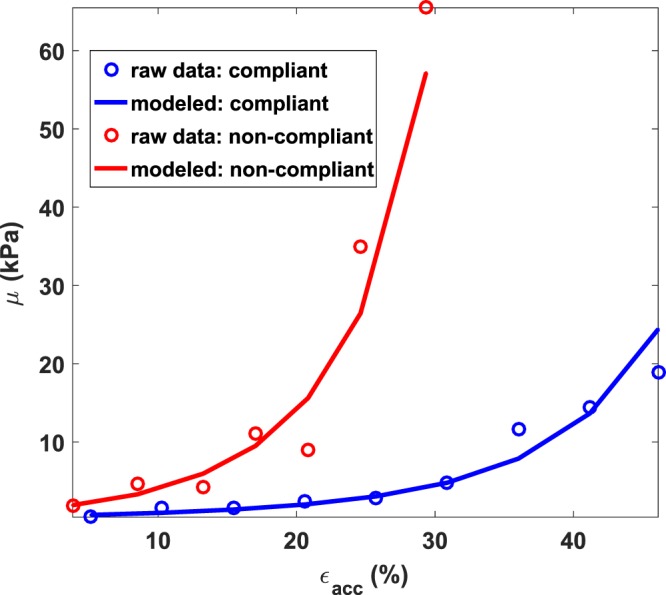
Elasticity versus accumulated strain in two patients with neurogenic bladders. Blue: diagnosed as compliant, red: diagnosed as non-compliant.

Figure 4 shows the raw data and corresponding model fits for two representative patients with compliant (blue) and non-compliant (red) bladders. The accoustoelasticity model fits closely for both cases with *r*^2^ values of 0.88 and 0.95 for compliant and non-compliant cases respectively. Additionally, the two cases presented different modulus-strain behaviors which translated into different nonlinearity parameter *A* (*A* = −10.5 kPa for compliant and *A* = −44.5 kPa for non-compliant). When applied in all cases, the mean fitting *r*^2^ value was 0.85 ± 0.11.

**Figure 5:**
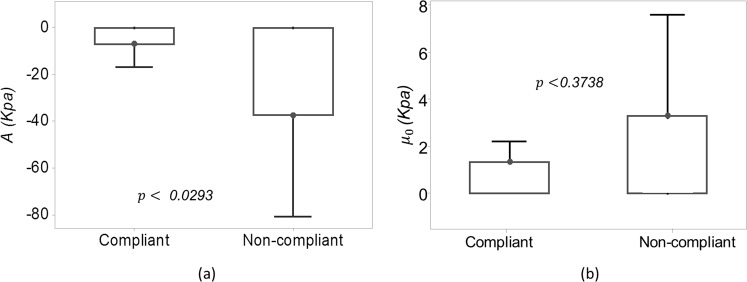
Box plots representing the distribution of (**a**) nonlinearity parameter $A$ and (**b**) predicted initial resting shear modulus ${\mu }_{0}$ for *in vivo* data.

Figure 5 shows the box-plot representation of the AE parameters *A* and *μ*_0_ in the two groups of patients. The median nonlinearity parameter *A* was −7.5 kPa in compliant and −33 kPa in non-compliant cases and this parameter was found to be significantly different in the two groups (*p* < 0.0293). The median predicted initial shear modulus *μ*_0_ was 1.00 kPa in compliant and 2.7 kPa in non-compliant cases. This parameter was not found to be significantly different in the two groups (p < 0.3738).

## Discussion

In this study we presented a noninvasive method for analyzing nonlinear elasticity properties of bladder tissue using AE theory. This technique requires measurement of the elasticity values at different loading levels and relating changes in modulus to the nonlinearity parameter *A*, initial modulus *μ*_0_ and incremental strain at each step. Measurement of the elasticity parameters of layered media requires selection of an appropriate model that accounts for geometric dispersions. For bladder tissue, a Lamb wave model had been shown to closely model wave dispersions along the wall^5^. This is the basis of UBV for noninvasive assessment of the bladder tissue mechanical properties. In this study, we used UBV method to capture the bladder tissue shear elasticity changes at different volumetric strain levels induced by increasing filling volumes. The AE model proposed by Gennisson *et al*.^12^, which related the speed of shear waves in pre-stressed media to the nonlinear parameter *A*, was properly modified to directly relate changes in the shear modulus (estimated by UBV) to the nonlinearity parameter *A*, initial modulus *μ*_0_ and incremental strain. We presented the utility of our method for measurement of nonlinearity parameter *A* and initial modulus *μ*_0_ via *ex vivo* and *in vivo* studies. Our *ex vivo* experiments showed the proposed method accurately modeled the nonlinear behavior in both intact and aberrant bladder samples. While *A* and *μ*_0_ were both higher in the formalin-treated samples compared to intact samples, the difference was only significant in the nonlinearity parameter *A*. We then applied our method in a group of patients with neurogenic bladders undergoing concurrent UDS and UBV studies. The proposed method accurately modeled changes in modulus as a function of accumulated strain via nonlinearity parameters (*r*^2^ = 0.85 ± 0.11). While both parameters *A* and *μ*_0_ were higher in the patients with non-compliant bladder compared to those with compliant bladder, only parameter *A* provided a significant difference (*p* < 0.0293). These findings are in accordance with pathophysiological changes of the bladder tissue in the neurogenic patients. Poor compliance leads to overgrowth of the fibrotic components in the bladder which may manifest as changes in the tissue mechanical properties such as the modulus of elasticity (second order) and nonlinear behavior (third and higher order elasticity coefficients). A number of factors may lead to inaccurate evaluation of the bladder wall nonlinearity using our method. Bladder filling can be challenging in patients with highly non-compliant bladder due to frequent leaking. This can lead to unwanted cycles of filling and voiding that may in turn undermine the accuracy of the estimated parameters under a unidirectional volumetric deformation. Additionally, to be able to perform UBV alongside UDS, the nonlinear behavior was only modeled using progressive volumetric deformation which may not properly reflect the hysteresis effect due to inherent viscosity. Another factor affecting the proposed method is related to the ultrasound imaging parameters used to locate the bladder wall as well as the tracking of transient Lamb waves, especially in bariatric population. Techniques such as harmonic and coded excitation ultrasound imaging can help overcome some of these problems. Another limitation of our study is related to a limited number of measurements which may lead to insufficient modulus-strain points required in the nonlinear inversion model. An improved system capable of acquiring continuous measurements of the modulus may help increasing the accuracy of the estimated parameters, especially in patients with frequency wall spams (e.g. patients with overactive bladders). Nevertheless, by quantifying the nonlinear behavior of the bladder wall, the preliminary results shown in this paper suggest that our method may provide invaluable information regarding the bladder tissue mechanical state that may be utilized in noninvasive diagnosis of non-compliant bladders. This method can be done non-invasively by starting with a full bladder and voiding in increment as described^6^. Additionally, due to noninvasive nature of our method, it can be used as a monitoring tool to assess the efficacy of treatments and prescribed medications.

## Conclusions

A new method for obtaining nonlinear elasticity associated with bladder tissue was presented. The method is based on combining the theory of AE for stressed materials and measurement of the shear elasticity on bladder wall using a Lamb wave model. Strong differentiation between intact and formalin treated *ex vivo* samples tissue samples was observed. When tested *in vivo*, the nonlinearity parameter was found to be significantly different in two groups of patients diagnosed as having compliant and non-compliant bladders. Hence, nonlinear elasticity parameter provided by our method, may provide additional information about the bladder tissue mechanical state which might have implications in the diagnosis of different diseases and conditions altering bladder functionality.

## Materials and Methods

### UBV Measurement of Shear Elasticity

In UBV, a focused acoustic radiation force is created on the bladder wall using an ultrasonic array transducer. Tissue motion in response to this force is then tracked using high speed imaging and Doppler-based speckle tracking algorithms^23^. Using 2-D Fourier transform, spatial-temporal tissue motion data are converted to k-space dispersion curves from which elasticity parameter are obtained using nonlinear fitting. To find the best fit, based on a homogeneity assumption, a group of dispersion curves are derived from the Lamb’s constitutive equation as (1)

1 $$4{k}_{L}^{3}\beta tanh(\beta h)={k}_{s}^{4}-{\beta }^{2}tanh({k}_{L}h)$$

where $\beta =\sqrt{{k}_{L}^{2}-{k}_{s}^{2}}$, ${k}_{L}=\omega /{c}_{p}$ is the Lamb wave number, *ω* is the angular frequency, *c*_*p*_ is the Lamb wave phase velocity, ${k}_{s}=\omega /\sqrt{\rho /\mu }$ is the wave number of shear component, *ρ* is the tissue density, *μ* is the complex modulus. Only storage shear modulus was used in this study.

### AE model Inversion

Given wall thickness and under a small curvature assumption, bladder wall radial strain can be used to approximate uniaxial strain at different volumes. In practice, bladder wall is difficult to identify in the ultrasound images at low filling volumes. Hence, UBV starts after accumulation of some initial filling volume. In unbounded medium, by considering shear wave displacements smaller than static compression, the nonlinear elastodynamic equation is written as

2 $$\rho {v}_{s}^{2}={\mu }_{0}-\sigma (\frac{A}{12{\mu }_{0}})$$

where ${v}_{s}^{2}$ is the shear velocity, *σ* is stress and μ_0_ is the shear modulus at stress-free (resting) condition. The left hand side of (2) can be also regarded as the instantaneous shear elasticity at each strain level. To adopt this model for pre-stressed layered media, the left hand side is replaced by the shear modulus estimate acquired using the Lamb wave model in (1) as

3 $${\mu }_{i}={\mu }_{0}-{\sigma }_{i}(\frac{A}{12{\mu }_{0}})$$

where *μ*_*i*_ is the measured modulus of elasticity at each strain level and *σ*_*i*_ is the total instantaneous stress imposed on the bladder wall. We follow the dynamic incremental loading scheme to predict the strain-stress response using a linear Hookean approximation at small compression levels as developed in^18^. Referring to $\sigma =E{\epsilon }$ and E ≈ 3*μ*, the Hooke’s law definition for the cumulative stress can be written as

4 $${\sigma }_{i}=\sum _{k=1}^{i}3{\mu }_{k}{\rm{\Delta }}{{\epsilon }}_{k}$$

Shear modulus at each compression step is then given by the following equation

5 $${\mu }_{i}={\mu }_{0}-[\sum _{k=1}^{i}3{\mu }_{k}{\rm{\Delta }}{{\epsilon }}_{k}](\frac{A}{12{\mu }_{0}})$$

where ${{\rm{\mu }}}_{0}$ is the predicted shear modulus at resting state and ${\rm{\Delta }}{\epsilon }_{{\rm{k}}}$ is the incremental strain generated due to compression at each step. Using UBV measurement of the elasticity at each volume ${\mu }_{i}$, and the corresponding strain ${\rm{\Delta }}{{\epsilon }}_{i}$, the projected initial resting elasticity ${\hat{\mu }}_{0}$, and the nonlinearity parameter $\hat{A}$, can be estimated by solving an unconstrained nonlinear least square problem. The instantaneous elasticity values can be arranged in a vector as ${\boldsymbol{\mu }}={[{\mu }_{1},{\mu }_{2},\ldots ,{\mu }_{N}]}^{{\rm{T}}}$ where ***μ*** can be expanded as

6 $${\boldsymbol{\mu }}=[\begin{array}{c}{\mu }_{0}-3{\mu }_{1}{\rm{\Delta }}{{\epsilon }}_{1}(\frac{A}{12{\mu }_{0}})\\ {\mu }_{0}-3({\mu }_{1}{\rm{\Delta }}{{\epsilon }}_{1}+{\mu }_{2}{\rm{\Delta }}{{\epsilon }}_{2})(\frac{A}{12{\mu }_{0}})\\ \vdots \\ {\mu }_{0}-[\sum _{k=1}^{N}3{\mu }_{k}{\rm{\Delta }}{{\epsilon }}_{k}\,](\frac{A}{12{\mu }_{0}})\end{array}]$$

Using (5) and solving for elements of ${\boldsymbol{\mu }}$, each entry can be written

7 $${\mu }_{i}=\frac{{\mu }_{0}-[{\sum }_{k=1}^{i-1}\,3{\mu }_{k}{\rm{\Delta }}{{\epsilon }}_{k}](\frac{A}{12{\mu }_{0}})}{1+3{\mu }_{i}{\rm{\Delta }}{{\epsilon }}_{i}(\frac{A}{12{\mu }_{0}})}\,i=1,2,\ldots ,N$$

Hence, in to order to estimate nonlinearity parameter $A$ and shear modulus at resting state ${\mu }_{0}$, a nonlinear least-square optimization problem can be formulated as

8 $$(\hat{A},{\hat{\mu }}_{0})=argmi{n}_{(A,{\mu }_{0})}{\Vert {{\boldsymbol{\mu }}}^{m}-{\boldsymbol{\mu }}\Vert }^{2}=argmi{n}_{(A,{\mu }_{0})}\sum _{i=1}^{N}{({\mu }_{i}^{m}-{\mu }_{i})}^{2}$$

### *Ex vivo* Study

**Figure 6:**
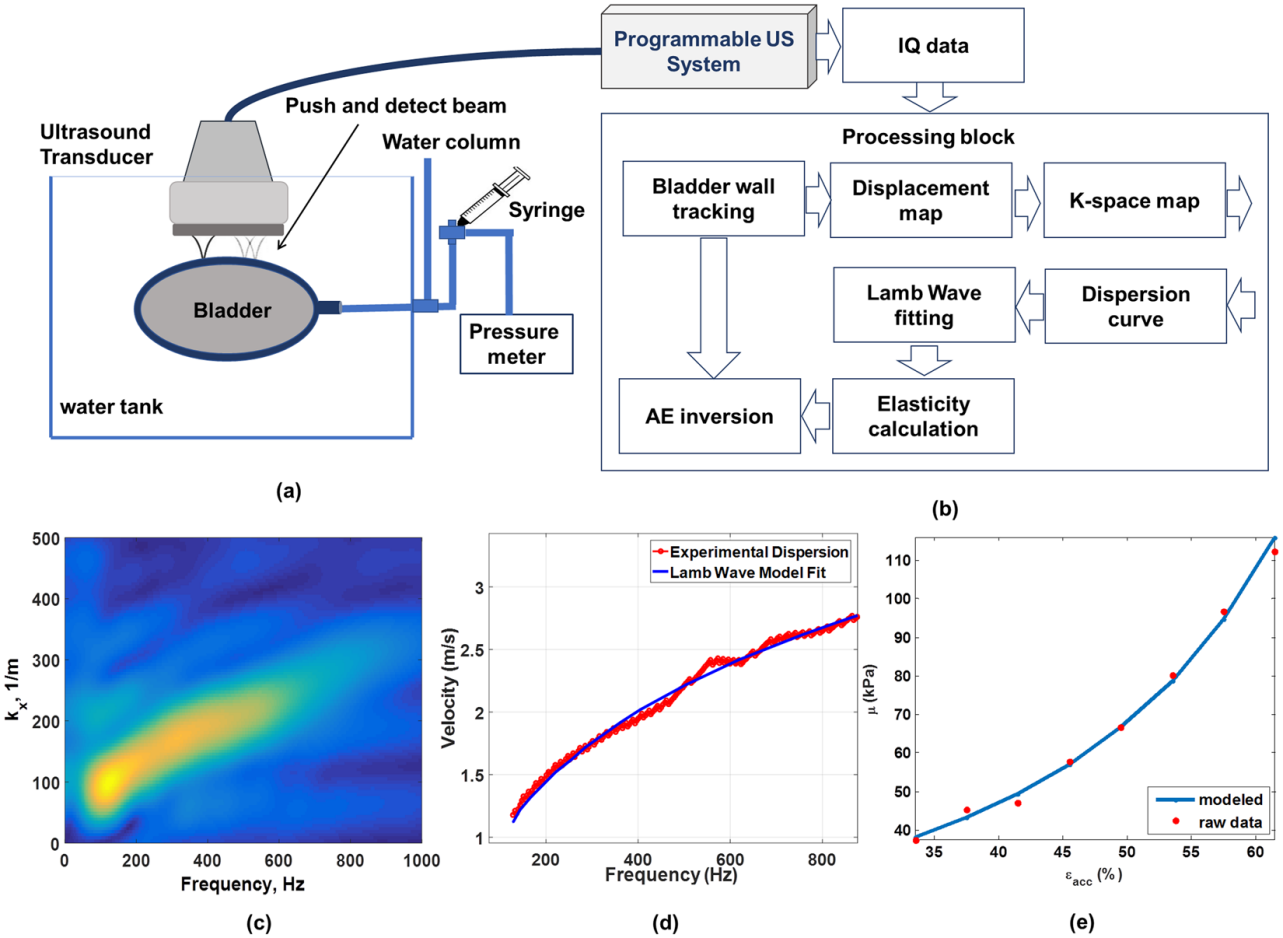
(**a**) *Ex*-*vivo* experimental setup for studying bladder AE parameters using UBV method, (**b**) processing chain, (**c**) k-space dispersion map related to tissue displacement along the bladder wall in response to acoustic radiation force, (**d**) experimental dispersion data and fitted curve using Lamb wave model and (**e**) experimental elasticity data as a function of strain level and fitted curve using AE model.

Freshly existed *ex vivo* porcine whole bladder samples were acquired from the Department of Surgery, Mayo Clinic Rochester, Minnesota under an approved Mayo Clinic IACUC protocol. All experiments are performed in accordance with the relevant guidelines and regulations of the Mayo Clinic Institutional Animal Care and Use Committee protocols. After blocking the ureters, the bladders were positioned in a water tank and were filled from urethra at incremental volume steps (see Fig. 6).At each volume, bladder elasticity was measured based on UBV method using a Verasonics programmable ultrasound machine (Verasonics, Kirkland, WA, USA) and a linear array transducer (L7-4, Philips, North America). Ultrasound imaging also provided accurate measurement of the bladder wall thickness from which incremental wall strain was calculated at each step. The minimum bladder thickness was set to 4 mm to avoid unrealistic accumulated strain values. The bladder samples were then soaked in formalin for about 24 hours. The entire procedure was repeated for the formalin-treated samples. The spatio-temporal map of the tissue motion in response to acoustic radiation force along the bladder wall (one direction) obtained from our *ex vivo* experiment on porcine bladder sample (see Fig. 6(a)). Two-dimensional Fourier transformation k-space dispersion data is then obtained as depicted in Fig. 6(b). The best Lamb wave fit is attained using ${{\rm{c}}}_{{\rm{p}}}({\rm{\omega }})={\rm{\omega }}/{\rm{k}}({\rm{\omega }})$ at each $({\rm{\omega }},{\rm{k}}({\rm{\omega }}))$ point along the ridge on the dispersion data. Figure 6(d) shows the analytic fit using Lamb model is in good agreement with the experimental data for a large range of frequencies. For both intact and aberrant bladders, AE parameters were estimated using equation (8) (see Fig. 6(e)). A Wilcoxon rank-sum test was used to measure the significance of changes in parameters *A* and ${\mu }_{0}$ in the two groups.

### *In vivo* Study

**Figure 7:**
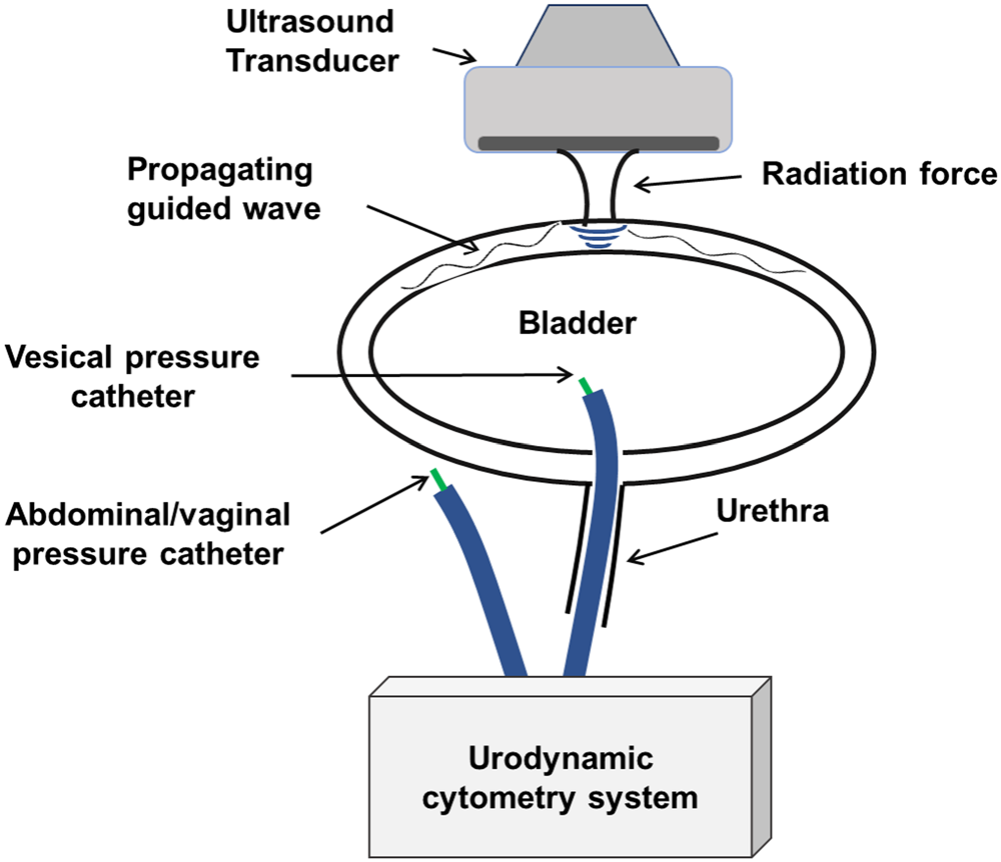
Schematic of concurrent UBV-UDS setup used in patient study.

Patient population for UBV study consisted patients with neurogenic bladder referring to Mayo Clinic urology department for routine UDS test. The study was approved by the Mayo Clinic institutional review board (IRB) and was Health Insurance Portability and Accountability Act (HIPAA) compliant. An informed written consent was obtained from patients prior to the examination. A programmable research ultrasound system (V1, Verasonics®, Redmond, Washington) and a curved linear array (C4-2, ATL/Philips, Bothell, WA) with center frequency of 2.5 MHz was used for the purposes of measurements. Urodynamic study Standard UDS consists of bladder catheterization through the urethra for gradual filling and simultaneous measurement of the intravesical pressure by a pressure sensor^6^. An abdominal pressure sensor is used to measure the pressure outside the bladder.The net detrusor pressure on the bladder wall is then calculated as the difference of the two pressure values. (See Fig. 7). UDS bladder filling was performed at 50 ml increments and at each volume three UBV measurements were performed. For each UBV acquisition, an ultrasound burst of 600–900 μs focused on the bladder wall was transmitted. The propagation of Lamb waves resulted from radiation force along the bladder wall was then tracked at 2500 frames per second using ultrasound plane-wave imaging with three angles of compounding^5^. Using recorded IQ data, particle displacement was calculated along the bladder wall curve using the autocorrelation technique^23^. Only storage modulus of shear elasticity was used in this study and it was acquired by fitting the experimental data to a wave dispersion curve obtained from (1). Using the variation in the thickness of bladder wall as results of the gradual changes in the volume, the incremental strain at each volume was calculated in each step. The minimum bladder thickness was set to 4 mm to avoid unrealistic accumulated strain values. The measured modulus of elasticity and strain were then used to obtain AE parameters A and ${\mu }_{0}$ using (8). Using the UDS results, volumetric changes and detrusor pressure changes were obtained for each patient. These values were then used to obtain the bladder detrusor compliance defined as the ratio of the maximum volumetric changes over the maximum detrusor pressure changes. Patients were grouped as compliant (compliance >40 cc/cmH_2_O) and non-compliant (compliance <40 cc/cmH_2_O) according to the guideline in^3^. Wilcoxon rank-sum test was used to measure the significance of changes in parameters A and ${\mu }_{0}$ in the two groups.

## Implementation

The approaches and processing described in this study have been implemented in Matlab® 2016(Mathworks Inc., Natick, MA).The off-line processing were carried out on a computer workstation (CPU @3.10 GHz Intel® Core™ i5, 16 GB RAM).
